# Supplementary material for: Content-rich biological network constructed by mining PubMed abstracts
Source: BMC Bioinformatics. 2004 Oct 8;5:147. doi: 10.1186/1471-2105-5-147 (PMC528731; doi:10.1186/1471-2105-5-147)
Supplement: Additional File 2 — The original results of the above study (non-essential files are deleted to keep the file size under the limit set by BMC bioinformatics). [file 1471-2105-5-147-S2.bz2 › chilibotAdditionalFile2/dip40/455ID10733106E10472/html/CCR3_CCL24.html]

 


 **CCR3** and **CCL24** 
  
Found 42 abstracts in PubMed, retrieved 40.  
 

 What does Google say? 
 PDF only 
| .edu only 

---

**Interactive relationship** (e.g. stimulation, inhibition, etc)

**Inhibitory relationship**- Identification of potent, selective non peptide CC chemokine receptor 3  [ **CCR3** ]  antagonist that inhibits eotaxin, eotaxin 2  [ **CCL24** ] , and monocyte chemotactic protein 4 induced eosinophil migration.  Ref: 10969084 J Biol Chem, 2000
- I TAC, Mig, and IP10 compete for the binding of eotaxin to  **CCR3**  bearing cells and inhibit migration and Ca 2 changes induced in such cells by stimulation with eotaxin, eotaxin 2  [ **CCL24** ] , MCP 2 monocyte chemottractant protein 2, MCP 3, MCP 4, and RANTES regulated on activation normal T cell expressed and secreted.  Ref: 11110785 J Biol Chem, 2001
- Moreover, the anti CC chemokine receptor 3  [ **CCR3** ]  monoclonal antibody, 7B11, was able to inhibit transient rise in the cytosolic Ca2 concentration and the release of reactive oxygen species following stimulation with eotaxin 2  [ **CCL24** ] .  Ref: 9692884 Eur J Immunol, 1998
**Neutral relationship**- Eotaxin and eotaxin 2  [ **CCL24** ] , acting through  **CCR3** , are potent eosinophil chemoattractants both in vitro and in animal models.  Ref: 12193745 J Immunol, 2002
- A linear peptide corresponding to the N terminal region of  **CCR3**  binds to eotaxin 2  [ **CCL24** ] , inducing concentration dependent chemical shift changes or line broadening of many residues.  Ref: 10913244 Biochemistry, 2000
- The human CC chemokine eotaxin 2  [ **CCL24** ]  is a specific agonist for the chemokine receptor  **CCR3**  and may play a role in the recruitment of eosinophils in allergic diseases and parasitic infections.  Ref: 10913244 Biochemistry, 2000
- The Eotaxin gene family Eotaxin1, Eotaxin2  [ **CCL24** ]  and Eotaxin3 recruits and activates  **CCR3**  bearing cells such as eosinophils, mast cells and Th2 lymphocytes that play a major role in allergic disorders.  Ref: 12761043 Hum Mol Genet< MedlineTA>Hum Mol Genet, 2000
- Our data suggest that eotaxin, eotaxin 2  [ **CCL24** ] , MCP 4, and RANTES induce eosinophil TEM via  **CCR3**  with varied potency and efficacy.  Ref: 10725746 J Immunol, 2000
- These data suggest that 1 eotaxin has a role in the early 6 h recruitment of eosinophils, while eotaxin 2  [ **CCL24** ]  and MCP 4 appear to be involved in later 24 h infiltration of these  **CCR3**  cells.  Ref: 10491000 J Immunol, 1999
- Eotaxin, eotaxin 2  [ **CCL24** ]  and eotaxin 3 have been identified as ligands that only activate CC chemokine receptor 3  [ **CCR3** ] .  Ref: 11286622 J Invest Dermatol, 2001
- Therefore, eotaxin 2  [ **CCL24** ]  represents a potent CC chemokine for human eosinophils activating chemotaxis related events, such as actin polymerization, and the respiratory burst via the  **CCR3** .  Ref: 9692884 Eur J Immunol, 1998
- Furthermore,  **CCR3**  transfected human embryonic kidney cells labeled with Fura 2AM exhibited a rapid rise in intracellular free calcium after stimulation with eotaxin, eotaxin 2  [ **CCL24** ] , or MCP 4, but not with MDC.  Ref: 10069890 J Allergy Clin Immunol, 1999

**Non-interactive relationship** (e.g. studied together, co-existance, homology, etc.)

- **CCR3**  ligands, i.e., regulated on activation normal T cell expressed and secreted RANTES, eotaxin, eotaxin 2  [ **CCL24** ] , and monocyte chemoattractant protein MCP 3, induced significant migration, while other chemokines showed no significant migration inducing ability.  Ref: 10505457 Allergy, 1999
- Stably transfected Cynomolgus  **CCR3**  bound human eotaxin CCL11 with similar kinetics Kd 240 pM and was responsive to human  **CCR3**  ligands eotaxin CCL11, eotaxin 2  [ **CCL24** ]   **CCL24** , and MCP4 CCL13 in Ca 2 mobilization and chemotaxis assays, thus provides a useful alternative species model system for the analysis of modulators of eotaxin  **CCR3**  induced signaling and activation.  Ref: 12421570 Cytokine, 2002
- Eosinophil chemotactic chemokines eotaxin, eotaxin 2  [ **CCL24** ] , RANTES, monocyte chemoattractant protein 3 MCP 3, and MCP 4, and C C chemokine receptor 3  [ **CCR3** ]  expression in bronchial biopsies from atopic and nonatopic Intrinsic asthmatics.  Ref: 10570327 J Immunol, 1999
- The existence of  **CCR3**  ligands other than eotaxin e.g., eotaxin 2  [ **CCL24** ]  may reflect the evolution of host counter measures to parasite defense systems.  Ref: 11086084 J Immunol, 2000
- Moreover, caspase I, IL 18, IL 12, and IFN gamma are found to be highly expressed at the skin lesion, occurring simultaneously with elevation of eotaxin 2  [ **CCL24** ]  and  **CCR3**  expression.  Ref: 11823539 J Immunol, 2002
- Pretreatment of cells with a  **CCR3**  blocking mAb, or PD98059, a MAP kinase inhibitor, prevented the eotaxin 2  [ **CCL24** ]  induced changes in eosinophil attachment.  Ref: 12034562 Am J Respir Cell Mol Biol, 2002
- In contrast, the  **CCR3**  agonists CCL11,  **CCL24**  and CCL26 are involved in the recruitment of cells such as eosinophils and basophils during Th2 responses.  Ref: 12884299 Eur J Immunol, 2003
- When eotaxin 2  [ **CCL24** ]  or other  **CCR3**  active chemokines were added to adherent eosinophils, it induced rapid and sustained eosinophil detachment from VCAM 1 in a concentration dependent manner.  Ref: 10946306 J Immunol, 2000
- Like eotaxin and eotaxin 2  [ **CCL24** ] , eotaxin 3 mediates its activity through  **CCR3** .  Ref: 12689946 Blood< MedlineTA>Blood, 2000
- **CCR3**  mediates in vitro chemotactic responses to the known ligands, eotaxin and eotaxin 2  [ **CCL24** ] , because the DC response to these chemokines is inhibited by  **CCR3**  specific mAbs.  Ref: 12218106 J Immunol, 2002
- Several CC chemokines including eotaxin CCL 11, eotaxin 2  [ **CCL24** ]  CCL 24  [ **CCL24** ] , RANTES CCL 5, and monocyte chemotactic protein 3 MCP 3, CCL 7 and 4 MCP 4, CCL 13 are potent eosinophil chemotactic and activating peptides acting through CC chemokine receptor 3  [ **CCR3** ]   **CCR3** .  Ref: 10969084 J Biol Chem, 2000
- AOP RANTES, RANTES, eotaxin and eotaxin 2  [ **CCL24** ] , but not Met RANTES, induced a downregulation of  **CCR3**  at 37 degrees C.  Ref: 11306977 Int Arch Allergy Immunol< MedlineTA>Int Arch Allergy Immunol, 2000
- The eotaxin group chemokines eotaxin, eotaxin 2  [ **CCL24** ] , and eotaxin 3 share only 35 41% sequence identity but are all agonists for the receptor  **CCR3** .  Ref: 12486712 Proteins, 2003
- Whereas no differences of  **CCR3**  downregulation and recycling after stimulation with AOP RANTES, RANTES, eotaxin and eotaxin 2  [ **CCL24** ]  were found there exists a distinct profile of activity with respect to the activation of the respiratory burst in human eosinophils.  Ref: 11306977 Int Arch Allergy Immunol< MedlineTA>Int Arch Allergy Immunol, 2003
- These ligands include the  **CCR3**  selective chemokines eotaxin and eotaxin 2  [ **CCL24** ]  and the more promiscuous CC chemokines, MCP 4, MCP 3, MCP 2 and RANTES.  Ref: 11306952 Int Arch Allergy Immunol< MedlineTA>Int Arch Allergy Immunol, 2003
- Compared with AC and NC, the numbers of EG2 cells and the cells expressing mRNA for eotaxin, eotaxin 2  [ **CCL24** ] , RANTES, MCP 3, MCP 4, and  **CCR3**  were significantly increased in AA and NAA p <.  Ref: 10570327 J Immunol, 1999
- In this study, we examined the effects of eotaxin 2  [ **CCL24** ] , a  **CCR3**  specific chemokine, on integrin mediated eosinophil adhesion to vascular cell adhesion molecule 1 VCAM 1, intercellular adhesion molecule 1 ICAM 1, or both using a parallel plate flow system.  Ref: 12034562 Am J Respir Cell Mol Biol, 2002
- The beta chemokine receptor  **CCR3** , which recognizes the ligands eotaxin, eotaxin 2  [ **CCL24** ] , monocyte chemotactic protein 3, MCP4, and RANTES, plays a central role in this process, and antagonists to this receptor could have potential therapeutic use in the treatment of allergy.  Ref: 10640766 J Immunol, 2000
- Interestingly, the novel  **CCR3**  specific chemokine, eotaxin 2  [ **CCL24** ] , showed the highest transcript levels.  Ref: 10415058 J Immunol, 1999
- We examined the expression of eotaxin, eotaxin 2  [ **CCL24** ] , RANTES, monocyte chemoattractant protein 3 MCP 3, MCP 4, and  **CCR3**  in the bronchial mucosa from atopic AA and nonatopic intrinsic.  Ref: 10570327 J Immunol, 1999
